# Case Series: Pleomorphic adenoma in minor salivary gland

**DOI:** 10.12688/f1000research.146682.1

**Published:** 2024-04-02

**Authors:** Meherzi Samia, Khbou Amin, Omri Rihab, Charfi Afifa

**Affiliations:** 1 Faculty of Medicine of Sousse, University of Sousse, Sousse, Tunisia; 2 ENT Department, Sidi Bouzid Hospital, Sidi Bouzid, Tunisia

**Keywords:** case report, salivary gland neoplasms, minor salivary gland, pleomorphic adenoma

## Abstract

Pleomorphic adenomas (PA) are the most prevalent benign salivary gland neoplasms. They may occur at any age, with a peak incidence between 40 and 60 years of age. They are more commonly observed in females (60%). These tumors can arise in both the major and minor salivary glands. Approximately 80% of these tumors are diagnosed in the parotid gland, whereas 10% arise in the minor salivary glands, mainly affecting the palates, followed by the lips and cheeks.

This report describes two cases of unusual lesions that were diagnosed as (PA) in the minor salivary glands in our department via a review of the relevant literature. The first case involved an 83-year-old man who presented with a slow-growing swelling on the right side of the upper lip, and the second case involved a 45-year-old woman who presented with a slow-growing lesion on the palate. The presence of PA was confirmed histopathologically after surgical resection.

Although relatively rare, PA is a benign lesion, the diagnosis of which must be known for appropriate therapeutic management.

## Introduction

Salivary gland tumors are relatively uncommon, accounting for 1-3% of head and neck cancers. Pleomorphic adenoma (PA) is a benign tumor that is most frequently diagnosed not only in the major salivary glands, namely the parotid gland (85% of cases) and submandibular gland (5% of cases), but also in the minor salivary glands (10% of cases). ^1^ The most common ectopic site of PA in the minor salivary glands is the palate, followed by the upper lip and oral mucosa. ^2^

Salivary gland neoplasms are a heterogeneous group of tumors with variable clinical appearances and histological features.

PA, also known as “Mixed tumor, salivary gland type,” receives its name from its wide pleomorphic architectural appearance and mixed histology that consists mainly of three components: an epithelial and a myoepithelial component within a mesenchymal stroma. ^3^ It is equipped with a fibrous capsule whose integrity must be conserved during surgical treatment to prevent recurrence. ^4^

PA is more common in middle-aged females.

Clinically, it is known to be a slow-developing, asymptomatic lesion, typically described as firm, well-delimited, and variable in diameter. Intraoral PAs are normally located in the submucosa with a firm or rubbery consistency. The mucosal lining remains intact, but ulcerations can be observed in some cases. ^5^

### Case 1

An 83-year-old man with no specific pathological history presented to the ENT Department at our hospital with a 2-year history of painless, slow-growing swelling on the right side of the upper lip.

**Figure 1.**
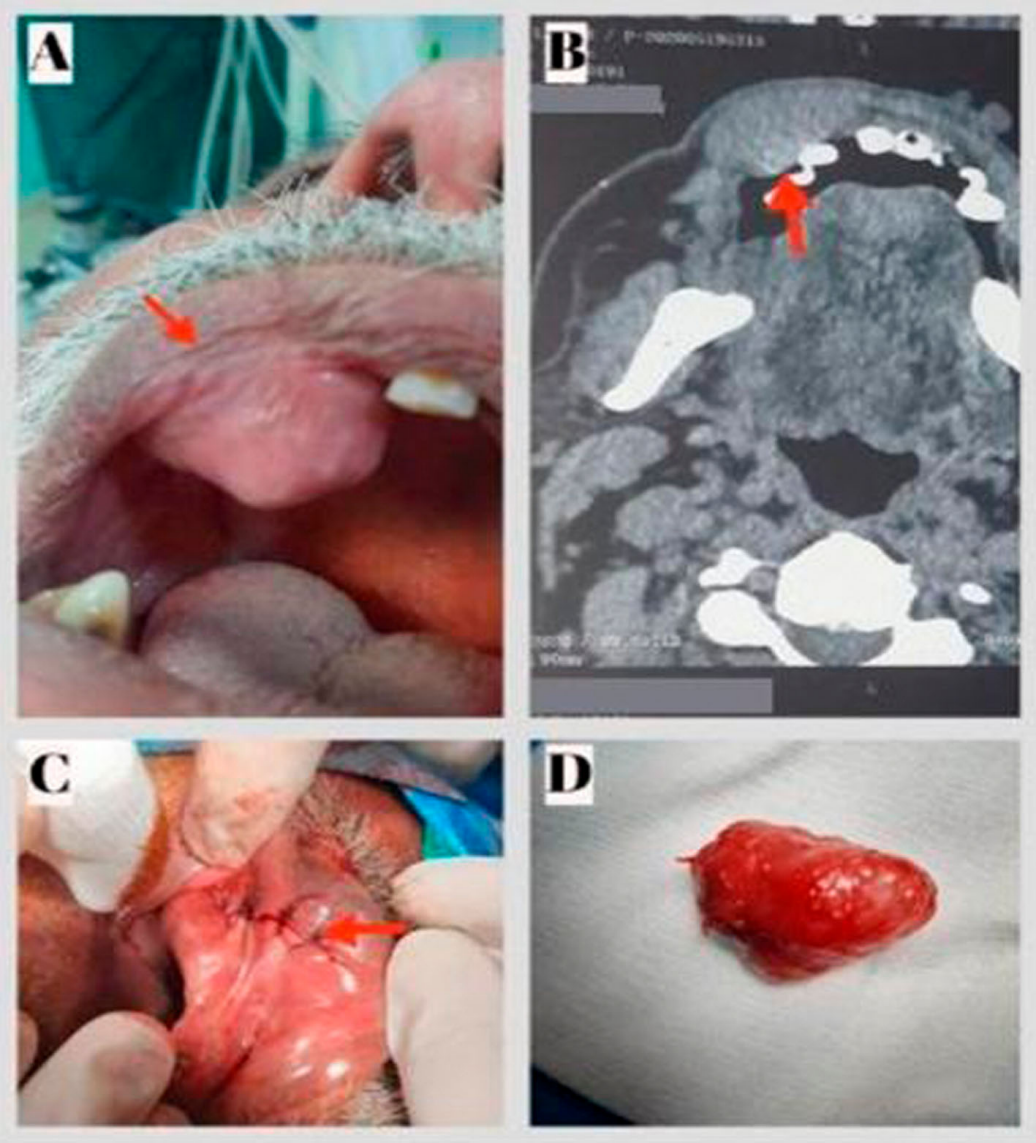
Pleomorphic adenoma of the upper lip. A: Preoperative view: Exposure of the mass in the palate. B: CT scan on axial section showing an oval well-defined lesion of the upper lip with homogeneous post-contrast enhancement. C: Postoperative view showing the site of the sutures. D: Excised specimen.

Clinical examination revealed a well-circumscribed, mobile, firm, and non-tender submucosal mass measuring 3×1.5 cm on the right side of the upper lip. The overlying mucosa appeared intact and smooth without bleeding on palpation ( Figure 1A). The lymph nodes of the head and neck were not enlarged.

Computed tomography (CT) showed an oval well-defined lesion of the upper lip measuring 3.2×1.7 cm with homogeneous post-contrast enhancement ( Figure 1B).

A total excision of the lesion via the sublabial approach was performed ( Figure 1C). The lesion was released from the surrounding tissue, and the mass appeared to be fully encapsulated ( Figure 1D). Histopathology of the resected tumor revealed the presence of a PA, a well-encapsulated soft tissue mass consisting of epithelial, myoepithelial, and stromal components ( Figure 3). The follow-up 24 months after surgery showed no abnormalities and no evidence of recurrence.

### Case 2

A 45-year-old woman with no medical history presented to our ENT department with a slow-growing painless nodular lesion in the palate that caused difficulty swallowing. Anamnesis revealed that the mass had appeared one year previously and had rapidly increased in size over the last three months.

**Figure 2.**
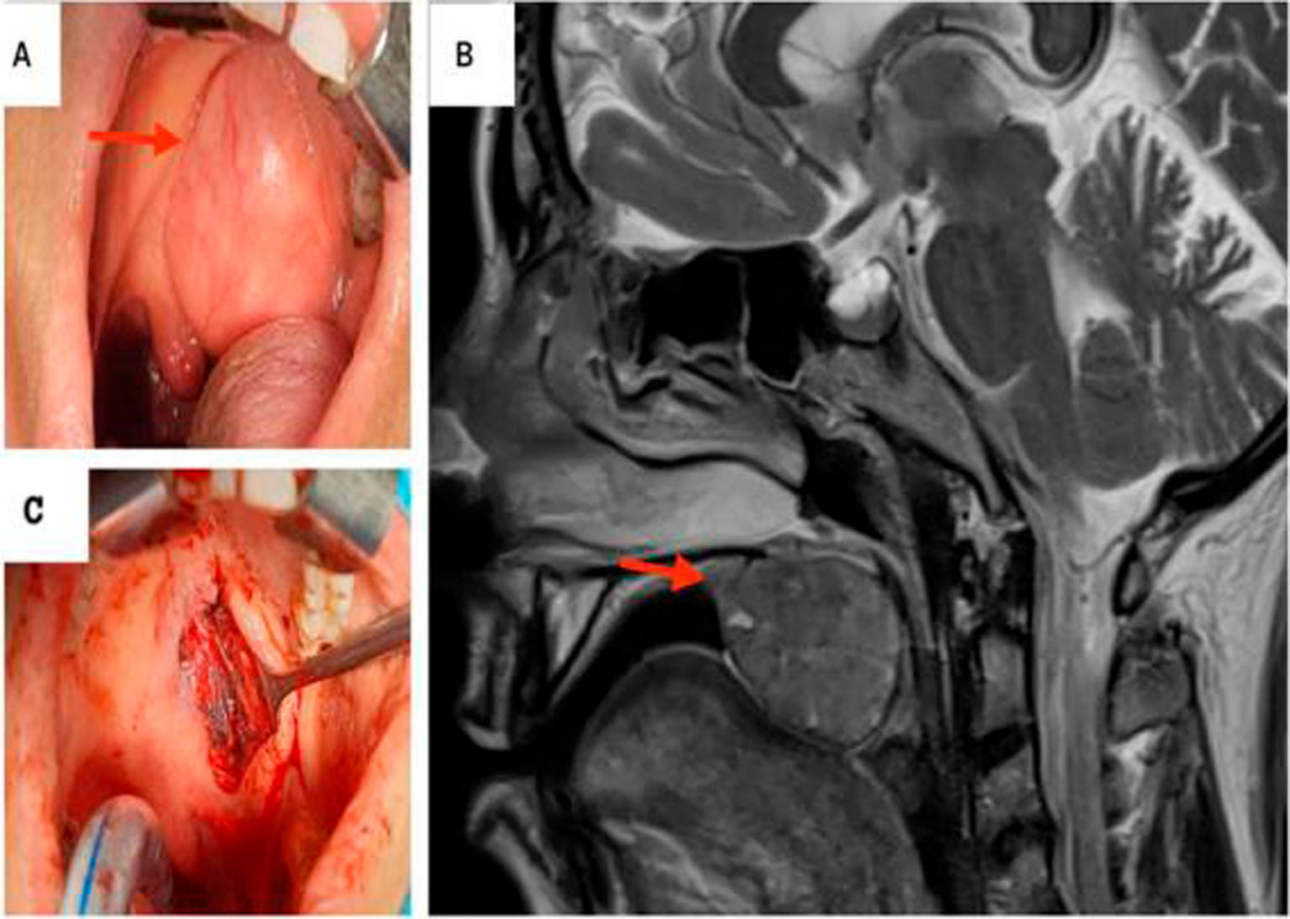
Pleomorphic adenoma of the palate. A: Preoperative view: Exposure of the mass in the palate. B: T2-weighted MRI showing a hyposignal ovoid well-defined mass within the right soft palate. C: Peroperative images: the defect post excision of the mass.

Intraoral examination revealed a unilocular, mobile, fibrous, endophytic nodule at the junction of the soft and hard palates, measuring approximately 4 cm in diameter. The nodule was well delimited, with a regular contour, smooth surface, and normal overlying mucosa color ( Figure 2A). No lymph node involvement was observed during the physical examination.

Magnetic resonance imaging (MRI) revealed an ovoid well-circumscribed encapsulated mass measuring 3.7 cm in size, within the midline of the soft palate to its right para-median side. The lesion extended backward to the oropharynx and forward in the left tonsillar pillar ( Figure 2B).

The mass was completely excised with safety margins via an intraoral approach ( Figure 2C).

**Figure 3.**
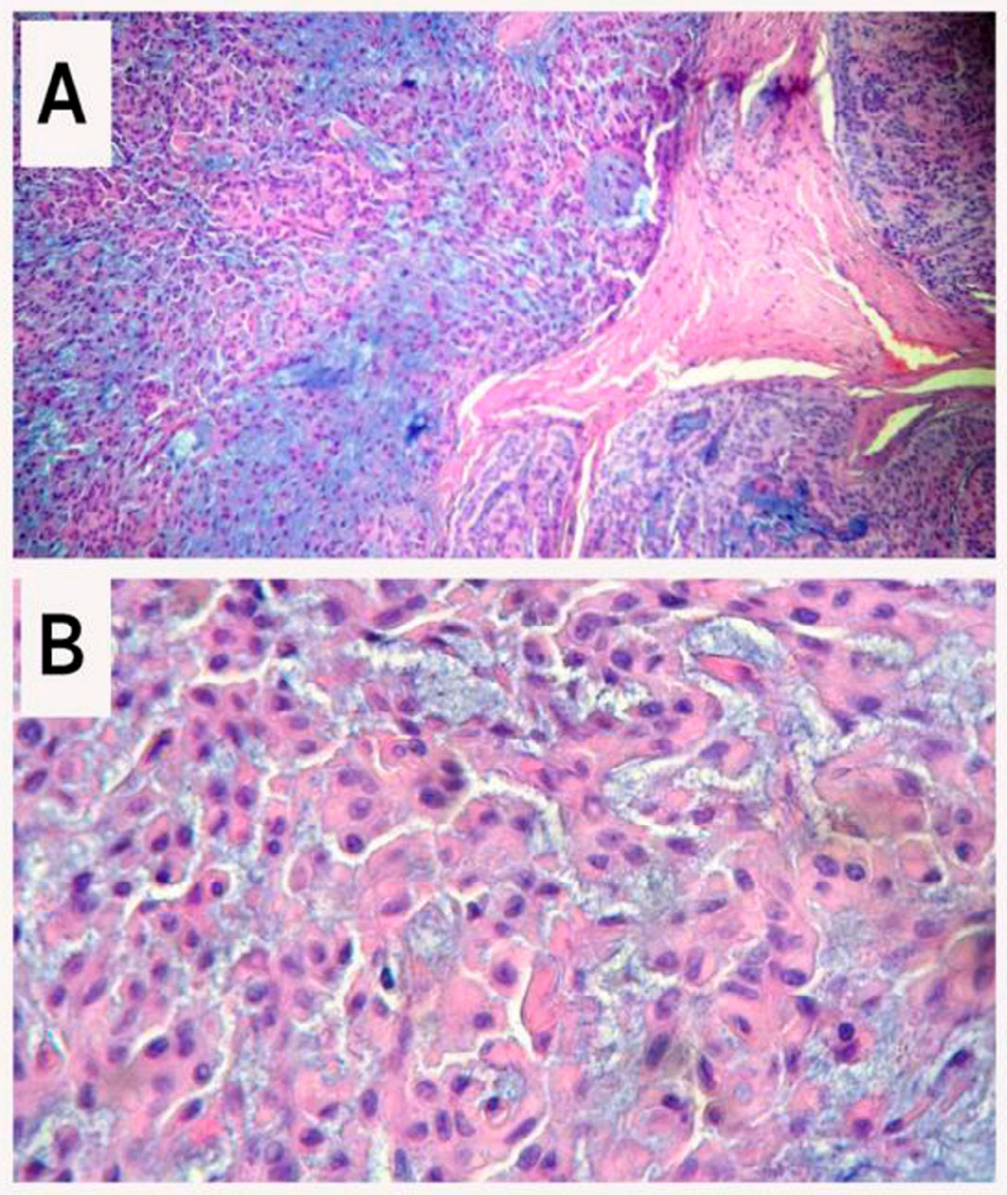
Photomicrograph showing epithelial salivary gland tumor. A: The tumor consists of three components: epithelial cells (right), myoepithelial cells (left), and myxochondroid tissue stroma (hematoxylin-eosin, 100×). B: Myoepithelial cells with epithelioid and plasmacytoid appearance (hematoxylin-eosin, 400×).

On final histopathological examination, the report suggested a PA ( Figure 3A & B).

An 18-month follow-up after surgery showed no abnormalities or evidence of recurrence.

## Discussion

Although malignant lesions are mostly diagnosed in minor salivary glands, benign lesions have also been reported. PA are the most common benign salivary gland neoplasms, representing approximately 3–10% of all neoplasms of the head and neck region. ^6^

They represent the most prevalent histopathological benign tumors diagnosed in both the major and minor salivary glands (50% of cases). Additionally, PA mainly affects the major salivary glands, particularly the parotids. The palate is the most prevalent intraoral site (42.8-68.8%), followed by the upper lip (10.1%) and cheek (5.5%). ^4^^,^^6^

It occurs in individuals of all ages but tends to affect more women than men, especially middle-aged individuals.

The present cases of PA in the minor salivary glands of the palate and the upper lip corroborate literature data showing a relatively low prevalence at these sites.

Regarding the epidemiological features of PA, gender, and age in our present cases, one of our two patients agreed with the literature, which shows that PA in the minor salivary glands may occur in individuals of all ages but most frequently affects women in their fourth to fifth decade of life, with a relevant mean age of 40-60 years.

However, in our first case, PA was diagnosed in an 83-year-old man, which does not match the relevant literature as neither the age of occurrence nor the sex of the patient was uncommon.

The clinicopathological features of our two cases concur with those reported in previous studies. In fact, PA generally presents as a mobile, slowly developing, painless, and firm swelling that does not cause any fixation or ulceration of the overlying mucosa with no lymph node involvement. ^7^

Histopathologically, PA is a complex mixed lesion consisting of both epithelial and myoepithelial components within a mucopolysaccharide stroma, organized predominantly in a duct-like pattern. They tend to have a fibrous capsule that separates the tumor from the surrounding tissues. The proportions of the different components can vary among individuals, parallel to changes in tumor consistency. ^6^^,^^8^^,^^9^

The need for complementary investigations into the management of PA in minor salivary glands depends on its localization. Indeed, a biopsy may be performed for additional oral sites.

However, if the diagnosis of PA is suspected in intraoral localizations, CT, ultrasonography, and optimal MRI are useful for studying the extent of the tumor and determining eventual bone involvement. ^3^^,^^10^

Biopsy is generally avoided because of the fear of the seedling. However, fine-needle aspiration is safe and recommended. ^11^

According to recent studies, the treatment of choice is wide local excision of the tumor with adequate margins, followed by histopathological examination to establish the final diagnosis. ^8^

Our therapeutic approach was consistent with the literature, as a total excision of the lesion via a sublabial approach was performed in the case of PA of the upper lip and via an intraoral approach for the patient who presented with PA of the palate.

Regular follow-up is necassary for patients with minor salivary gland neoplasms, not only due to their heightened tendency toward local recurrence but also because of their malignant potential. ^12^^,^^13^

## Conclusion

The diverse presentations of pleomorphic adenomas make diagnosis complicated and challenging. PAs of the minor salivary glands are rare neoplasms. While their occurrence in the minor salivary glands is uncommon, obtaining the correct diagnosis as early as possible is essential because early initiation of appropriate treatment allows for effective management and improves patient prognosis. Complete wide local surgical excision is the treatment of choice. Patients should be followed up for a longer period to detect late recurrences.

## Ethics and consent

Written informed consent was obtained from both patients for publication and accompanying images.

## Author contribution

Samia Meherzi: writing, review and editing

Rihab Omri: conceptualization, writing

Amin Khbou: conceptualization, preparation

Afifa charfi: review

## Data Availability

All data underlying the results are available as part of the article and no additional source data are required.
