# Supplementary material for: Case Series: Pleomorphic adenoma in minor salivary gland
Source: F1000Res. 2024 Jun 28;13:243. Originally published 2024 Apr 2. [Version 2] doi: 10.12688/f1000research.146682.2 (PMC11237821; doi:10.12688/f1000research.146682.2)
Supplement: Peer review history

# Reviewer report: Reviewer response for version 2

**DOI:** 10.5256/f1000research.168319.r296834

**Published:** 2024-07-10

**Authors:** Atsuto Katano

**Affiliations:** 1 University of Tokyo Hospital, Tokyo, Japan

I believe the article has improved significantly by incorporating the peer review comments.

Are enough details provided of any physical examination and diagnostic tests, treatment given and outcomes?

Yes

Is the background of the cases’ history and progression described in sufficient detail?

Yes

Is sufficient discussion included of the importance of the findings and their relevance to future understanding of disease processes, diagnosis or treatment?

No

Is the conclusion balanced and justified on the basis of the findings?

Yes

Reviewer Expertise:

Clinical oncolgy

I confirm that I have read this submission and believe that I have an appropriate level of expertise to confirm that it is of an acceptable scientific standard.

# Reviewer report: Reviewer response for version 1

**DOI:** 10.5256/f1000research.160795.r262620

**Published:** 2024-05-07

**Authors:** Dorra Chiboub

**Affiliations:** 1 Tunis El Manar University, Tunis, Tunisia

- The objective of the article must be clarified in the introduction
- Specify patients' habits (examples: tobacco consumption, alcohol)
- Physical examination must include, for both cases, the performance of a nasofibroscopy and the result of this examination to look for possible associated lesions, especially malignant
- Put more recent references

Are enough details provided of any physical examination and diagnostic tests, treatment given and outcomes?

Partly

Is the background of the cases’ history and progression described in sufficient detail?

Yes

Is sufficient discussion included of the importance of the findings and their relevance to future understanding of disease processes, diagnosis or treatment?

Yes

Is the conclusion balanced and justified on the basis of the findings?

Yes

Reviewer Expertise:

cervical surgery

I confirm that I have read this submission and believe that I have an appropriate level of expertise to confirm that it is of an acceptable scientific standard.

# Response

**Published:** 2024-06-24

**Authors:** Meherzi Samia

**Affiliations:** university of sousse, faculty of medecine of sousse, Tunisia, Tunisia

In response to your valuable feedback, we've strengthened the introduction by explicitly stating the study objective. Additionally, we've provided clearer details about patient habits and endoscopic findings. Finally, the manuscript now includes recent references to support the research and enhance its credibility.

# Reviewer report: Reviewer response for version 1

**DOI:** 10.5256/f1000research.160795.r262621

**Published:** 2024-04-13

**Authors:** Atsuto Katano

**Affiliations:** 1 University of Tokyo Hospital, Tokyo, Japan

Major Points:

 i ) In the introduction section, there is no indication of what knowledge gap or research question the study aims to address. A well-defined research objective or question would enhance the significance of the study.

 ii) In the introduction section, although the text briefly mentions demographic characteristics, it does not provide a broader epidemiological context. For instance, there is no discussion of incidence trends, geographic variations, or associated risk factors. Providing this information would enhance the reader's understanding of the significance of studying pleomorphic adenoma (PA).

 iii) The histopathological description of the pleomorphic adenoma (PA) is brief and lacks detailed information on specific immunohistochemistry, histopathological, and/or genomic features that are characteristic of PA.

 iv) In the discussion section, there is no mention of future research directions or unresolved questions arising from the study's findings.

 v) In the conclusion section, the author mentioned the patient prognosis of pleomorphic adenoma (PA). They should discuss the patient prognosis of PA in the discussion section, including overall survival (OS) and/or progression-free survival (PFS).

 Minor Points:

 i) Several statements lack proper citation to support claims made in main text.

 e.g.

 - PA is more common in middle-aged females.

 - It occurs in individuals of all ages but tends to affect more women than men, especially middle-aged individuals.

 - most frequently affects women in their fourth to fifth decade of life, with a relevant mean age of 40-60 years.

 ii) What is "ENT Department," which descrtbed in Case 1.

Are enough details provided of any physical examination and diagnostic tests, treatment given and outcomes?

Yes

Is the background of the cases’ history and progression described in sufficient detail?

Yes

Is sufficient discussion included of the importance of the findings and their relevance to future understanding of disease processes, diagnosis or treatment?

No

Is the conclusion balanced and justified on the basis of the findings?

Yes

Reviewer Expertise:

Clinical oncolgy

I confirm that I have read this submission and believe that I have an appropriate level of expertise to confirm that it is of an acceptable scientific standard, however I have significant reservations, as outlined above.

# Response

**Published:** 2024-06-24

**Authors:** Meherzi Samia

**Affiliations:** university of sousse, faculty of medecine of sousse, Tunisia, Tunisia

We've significantly strengthened the manuscript by incorporating your valuable feedback. The introduction now provides a clear objective and relevant epidemiological context. Additionally, we've enriched the discussion of pleomorphic adenoma (PA) with detailed histological features, immunohistochemistry (IHC) analysis, and a comprehensive exploration of prognosis.
